# Genome sequence of *Pseudomonas* sp. MAHUQ-62 isolated from a pumpkin garden located in Anseong, South Korea

**DOI:** 10.1128/mra.00915-25

**Published:** 2025-09-30

**Authors:** Md. Amdadul Huq, Md. Mominul Islam, Md. Shahedur Rahman

**Affiliations:** 1 Department of Life Sciences, College of BioNano Technology, Gachon University 65440, Seongnam, South Korea; 2 Research & Development Institute, Nano. Project Group. Moorim P&P Co., Ltd., Ulsan, South Korea; 3 Department of Genetic Engineering and Biotechnology, Jashore University of Science and Technology 421984, Jashore, Bangladesh; DOE Joint Genome Institute, Berkeley, California, USA

**Keywords:** genome sequence, *Pseudomonas* sp. MAHUQ-62, pumpkin garden

## Abstract

This study reports the draft genome sequence of *Pseudomonas* sp. MAHUQ-62, a bacterial strain isolated from the soil sample of a pumpkin garden located in Anseong, South Korea, in 2020. The genome consists of 6,038,011 base pairs assembled into 57 contigs, encoding 5,478 predicted protein-coding genes.

## ANNOUNCEMENT

The genus *Pseudomonas*, first described by Migula in 1894 (1), currently comprises 355 validly published species (2) that have been isolated from diverse environments worldwide, including soil, animals, plants, fresh and saline water, and clinical specimens (3–8). During investigations on bacterial biodiversity in a pumpkin garden, a new strain of the genus *Pseudomonas*, designated *Pseudomonas* sp. MAHUQ-62 was isolated, and its draft genome assembly is presented in this study. Sequencing bacterial genomes serves as a valuable technique with broad applications in medicine, public health, and evolutionary research (9–13).

Strain MAHUQ-62 was isolated in 2020 from the soil sample of a pumpkin garden located in Anseong, South Korea (37° 00′ 45″ N, 127° 22′ 25″ E). One gram of soil sample was suspended in 9 mL of sterile 0.85% (w/v) NaCl solution. The suspension was serially diluted up to a 10^−6^ dilution, and 100 µL of the individual dilution was spread onto TSA plates. Then, the plates were placed in a 28°C incubator for three days. Single colonies were purified by repeated streaking on fresh TSA plates. Genomic DNA was extracted from the culture of a single colony grown in TSB for 24 h using the Solg Genomic DNA Prep kit (Solgent, Republic of Korea), according to the manufacturer’s protocol. Whole-genome sequencing was performed at the World Data Centre for Microorganisms, The Institute of Microbiology of the Chinese Academy of Sciences, Beijing 100101, China. The DNA fragments were size-selected using AMPure XP beads to isolate those within the desired range, which is typically around 250 base pairs (bp) for standard Illumina sequencing libraries. The library for sequencing was prepared using the Nextera XT DNA Library Prep Kit (Illumina, San Diego, USA). After library preparation, sequencing was carried out using the Illumina HiSeq 3000 platform with paired-end 2×150 bp reads following the standard protocol provided by the manufacturer. Illumina’s bcl2fastq software version 2.20.0 (https://support.illumina.com/sequencing/sequencing_software/bcl2fastq-conversion-software.html) was utilized with default parameters to demultiplex the raw reads and convert them into a FASTQ format. For further downstream analysis, raw paired-end reads from the HiSeq platform were used. Reads were processed with Trimmomatic version 0.38 (14). The genome sequence was assembled by the SOAPdenovo v2.04 assembler (15). The genome was annotated with the NCBI Prokaryotic Genome Annotation Pipeline version 6.3 (16–18). The strain was identified using a whole genome-based taxonomic analysis in the Type (Strain) Genome Server (19). Default parameters were used for all software unless otherwise specified. The genome-to-genome distance bioinformatics tool (20) was used to measure *in silico* DNA-DNA hybridization values. The best matches to the MAHUQ-62 genome are the type strain *Pseudomonas resinovorans* DSM 21078 (accession: GCA_000423545) and *Pseudomonas lalkuanensi* MCC 3792 (accession: GCA_008807375) with *in silico* DNA-DNA hybridization values of 34.3 and 33.9%, respectively.

The number of reads generated by genome sequencing was 6,455,984. The assembly contains 57 contigs with a coverage of 139×. The genome of *Pseudomonas* sp. MAHUQ-62 is 6,038,011 bp long with a GC content of 63.0%. A total of 5,634 genes were predicted, of which 5,478 are protein-coding genes. The details of genomic features are presented in Table 1.

**TABLE 1:** Genomic features of *Pseudomonas* sp. MAHUQ-62

Description of source	
Location	Anseong, South Korea
Time	2020
Type	Soil of pumpkin garden
Sequencing summary	
Coverage	139×
Total bases	6,038,011
GC content	63.0%
Assembly report	
Contigs	57
Contig *L*_50_	7
Contig *N*_50_	240.2 kb
Genome length	6 Mb
Annotation report	
Genes (total)	5,634
CDSs (total)	5,562
CDSs (with protein)	5,478
ncRNAs	4
tRNA	62

## Data Availability

The draft genome sequence of Pseudomonas sp. MAHUQ-62 was deposited in GenBank under accession number JBPZTF000000000. The raw reads are available in SRA under accession number SRR33604968. The version described in this paper is the first version, JBPZTF000000000.1.
